# In this month’s Bulletin

**DOI:** 10.2471/BLT.20.000520

**Published:** 2020-05-01

In the editorial section Chalapati Rao (298) emphasizes the need for accurate certification of cause-of-death. Bernardo Sousa-Pinto et al. (299) describe how the rates of testing for COVID-19 affect hospitalizations. 

In the news section, Tatum Anderson (302–303) reports on the manufacturing processes needed to meet global demand once an effective vaccine for SARS-CoV-2 is developed. Amesh Adalja talks to Gary Humphreys (304–305) about the lessons being learned in the COVID-19 epidemic.

## Botswana

### Community health worker guidelines

Stephanie Watson-Grant et al. (370–372) identify an evaluation gap.

## China

### Antibiotic use in livestock

Yanhong Jessika Hu & Benjamin John Cowling (360–361) note reduction measures.

## Ethiopia, South Africa

### Multi-drug resistant tuberculosis

Jason J Madan et al. (306–314) provide an economic evaluation of short treatment regimens.

## Sierra Leone

### Countering Ebola 

Mohamed F Jalloh et al. (330–340) study behavior change in an outbreak.

## Slovenia

### Reaching each community

Anne S Johansen et al. (353–359) trace developments in primary health care.

## Global

### Herpes simplex virus

Charlotte James et al. (315–329) estimate global incidence and prevalence 

### Emergency care in low and middle-income countries

Kalin Werner et al. (341–352) review the evidence on cost-effectiveness of interventions

### Monitoring health effects of volcanic eruptions 

William Mueller et al. (362–364) propose standard epidemiological protocols. 

### Noma as a neglected disease

Alexandra Caulfield & Tobias Alfvén (365–366) argue for better prevention and treatment.

### Noise levels in concert venues

Elizabeth Francis Beach et al. (367–369) review measures to reduce risk of hearing damage.

